# Safety of intravenous anakinra in COVID-19 with evidence of hyperinflammation, a case series

**DOI:** 10.1093/rap/rkaa040

**Published:** 2020-08-04

**Authors:** Kristina E N Clark, Oliver Collas, Helen Lachmann, Animesh Singh, Jim Buckley, Sanjay Bhagani

**Affiliations:** r1 Department for Rheumatology and Connective Tissue Diseases, University College London; r2 Department of Intensive Care, Royal Free Hospital London NHS Foundation Trust; r3 UK National Amyloidosis Centre, University College London; r4 Department of Rheumatology; r5 Department of Infectious Diseases, Royal Free Hospital London NHS Foundation Trust, London, UK

**Keywords:** COVID-19, haemophagocytic lymphohistiocytosis, anakinra, hyperinflammation

## Abstract

**Objectives:**

Anakinra is a selective IL-1 inhibitor, which has been used in the context of secondary haemophagocytic lymphohistiocytosis. Although usually given in the s.c. form, previous anecdotal reports have emphasized its utility when given i.v. Our aim is to report our experience on the beneficial effects of anakinra i.v. in patients with SARS-CoV-2 and evidence of hyperinflammation.

**Methods:**

We report four patients with severe COVID-19 infection requiring intensive care admission and ventilatory support.

**Results:**

All four patients showed evidence of deterioration, with hyperferritinaemia and increasing oxygen requirements and with superadded bacterial infections. Upon commencement of anakinra i.v., there was subsequent improvement in the patients clinically, with reduction in ventilatory support and inotropic support, and biochemically, with rapid improvement in inflammatory markers.

**Conclusion:**

Anakinra is safe to use i.v. in patients with COVID-19 and evidence of superadded bacterial infection. Although its utility has not been confirmed in a randomized trial, current research in the COVID-19 pandemic aims to establish the utility of immunosuppression, including IL-1 blockade, on the outcomes of patients with moderate to severe disease. Our case series supports its use in patients with severe, life-threatening COVID-19 and evidence of hyperinflammation.

**Key messages**

- Anakinra is safe in severe COVID-19 infections with evidence of superadded bacterial infection.
- Anakinra i.v. results in clinical and biochemical improvement in severe COVID-19 requiring ventilatory support.
- Anakinra should be considered in the arsenal of treatment options for COVID-19.

## Introduction

Anakinra is a recombinant IL-1 receptor antagonist originally marketed for use in RA. It has increasingly found off-label use in patients with haemophagocytic lymphohistiocytosis (HLH). Efficacy data come predominantly from case series [1, 2], where it achieves disease remission, normalization of laboratory abnormalities and resolution of pyrexia from cytokine storm [3, 4], even in the context of sepsis. Traditionally given s.c., the i.v. form is safe at doses ≤10 mg/kg [5]. Despite the limited evidence, consensus guidelines support its use in the treatment algorithm of cytokine storm [6].

COVID-19 typically presents with fever, dry cough and dyspnoea, although the array of symptoms is wide. The mean time lag from symptom onset to dyspnoea is 5–8 days, and to ARDS it is 8–14 days [7, 8]. ARDS affects 8–19% of patients [3], and these patients have increased risk of cytokine storm and progressive multi-organ damage. Severe COVID-19 is more common in older patients and in those with certain co-morbidities, including obesity, diabetes and cardiovascular disease [9].

Severe COVID-19 is characterized by prominent alveolar damage, with focal reactive hyperplasia of pneumocytes, patchy inflammatory cellular infiltration and intravascular thrombosis. Key pathological cells include CD4^+^ and CD8^+^ T cells and macrophages. The cytokine profile is similar to HLH, with increased IL-6, IL-1β, IL-10, GM-CSF, IFN-γ, macrophage inflammatory protein 1-α, monocyte chemoattractant protein-1 and TNF-α [3, 10, 11].

SARS-CoV2 evades a number of immune system recognition points that usually initiate virus-mediated immunity. The activation of innate immune cells by infected macrophages results in the expression of pro-inflammatory IL-1, IL-6 and TNF-α production through the nuclear factor-κB pathway. The concentrations of these cytokines continue to increase via a process of auto-amplification, and recruit adaptive immune cells [12].

Aberrant CD4^+^ T-cell activation releases IFN-γ and GM-CSF. The GM-CSF results in increased CD14^+^CD16^+^ inflammatory monocyte subsets, a subset rarely seen at significant levels in health [13]. This subset of monocytes expresses increased levels of IL-6, which are likely to be responsible for the acceleration and progression of a systemic ihinflammatory response.

IL-1 and related pro-inflammatory pathways intertwined with aberrant T-cell responses play a crucial role in disease severity [14]. Elevated ferritin and IL-6 concentrations are correlates with mortality [15]. We define hyperinflammation by this systemic inflammatory response, which has strong similarities to that seen in cytokine release syndrome. One current treatment strategy is to control hyperinflammation with targeted immunosuppression.

We describe a cohort of patients with severe SARS-CoV-2 infection admitted to the intensive care unit (ICU) during the pandemic, with elevated ferritins between 4000–30 000 µg/l. All four patients were diagnosed with COVID-19 and required ventilatory support. These patients all showed evidence of hyperinflammation with raised inflammatory markers and CRP and were given anakinra i.v., with safe and successful use, suggesting the potential benefit of IL-1 blockade in this subgroup of patients with confirmed COVID-19.

## Case series

### Case 1

A 30-year-old man presented with a background of end-stage renal failure secondary to birth asphyxia and with a donation after brainstem death renal transplant (baseline creatinine 290 µmol/l), maintained on sirolimus and tacrolimus. This was the patient’s second transplant, the first being complicated by graft rejection, microangiopathic haemolytic anaemia and requiring graft nephrectomy in 2013. The current transplant had taken place >1 year before the current presentation, of which he had had one episode of BK viral nephropathy treated with CSs. He had been stable after this, with no significant infections. He presented with a 14-day history of feeling unwell with fevers, and a 5-day history of a cough, sore throat and difficulty breathing.

Initial oxygen saturation was 80% on room air, which improved to 100% on 35% oxygen via a venturi face mask. His blood pressure was 127/73 mmHg, heart rate 95 beats/min, temperature 38°C and respiratory rate 21 breaths/min. Chest X-ray (CXR) showed bilateral patchy consolidation, and nasopharyngeal swab (NPS) confirmed SARS-CoV-2. Blood tests revealed ferritin of 24617 µg/l, creatinine 519 µmol/l and CRP 92 mg/l.

**Fig. 1:**
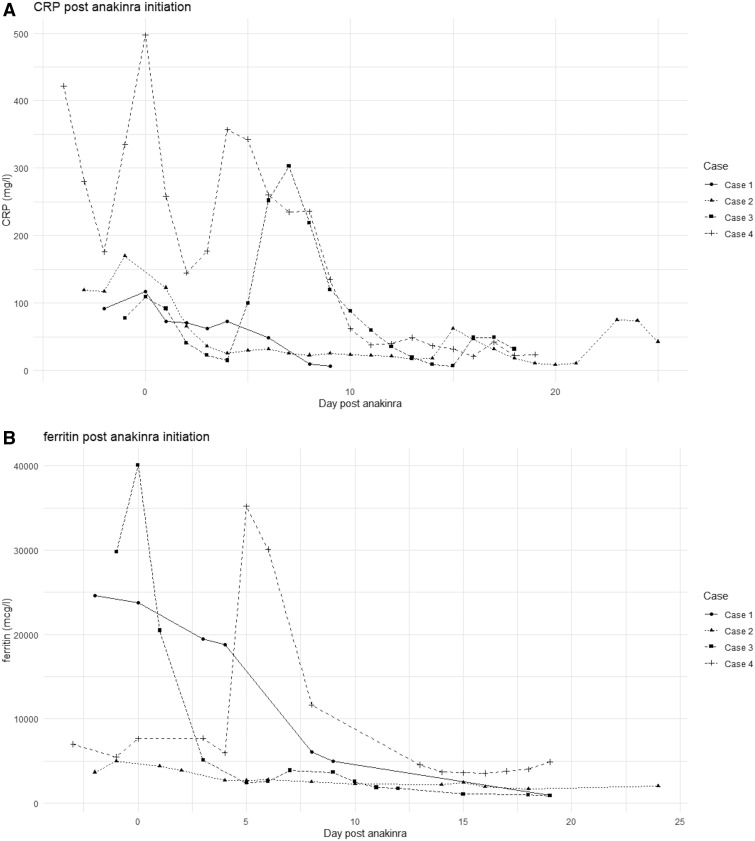
Graphs showing serum ferritin and CRP concentrations after initiation of anakinra for all four cases (**A**) Ferritin concentration (in micrograms per litre). (**B**) CRP concentration (in milligrams per litre).

Oxygen was titrated to keep his saturations >94%, and he was initiated on co-amoxiclav. After 2 days, with increasing oxygen requirements and progressive CXR consolidation, he was transferred to the ICU, where he was commenced on continuous positive airway pressure. The sirolimus was stopped, but he remained on tacrolimus. Ferritin at the time was 23 788 µg/l, CRP 73 mg/l and procalcitonin 17.27 μg/l (normal range <0.5 μg/l). Microscopy from blood cultures and urine cultures was negative, and CMV and EBV viraemias remained very low level. Given the increasing oxygen requirement and persistent inflammatory state, anakinra 200 mg i.v. was initiated (Table 1). A marked improvement in ferritin was seen within 2 days; CRP steadily decreased (Fig. 1), creatinine improved, and his respiratory effort stabilized. After 3 days of continuous positive airway pressure, he was weaned off and stepped down to the ward. Anakinra i.v. was stopped on day 10, when the ferritin was 4969 µg/l. He was discharged on day 12 with a creatinine of 371 µmol/l and CRP 7 mg/l and remained on tacrolimus.

**Table 1:** Table to highlight different medications used during the care of each case

	Case 1	Case 2	Case 3	Case 4
Before admission	Sirolimus 3 mg	Tacrolimus 5 mg BD	Rituximab 500 mg	Prednisolone 10 mg
		MMF 500 mg BD		
	Tacrolimus 2/1 mg	Prednisolone 5 mg OD		
Anakinra dose	200 mg OD	200 mg OD	200 mg BD	200 mg OD
Antibiotics and antifungals	Ceftriaxone	Ceftriaxone	Ceftriaxone	Ceftriaxone
		Teicoplanin	Meropenem	Gentamicin
			Ambisome	Temocillin
				Teicoplanin
				Meropenem
				Caspofungin

BD: twice a day; OD: once a day.

### Case 2

A 48-year-old man was admitted 11 days after having a donation after brainstem death renal transplant for end-stage renal failure secondary to IgA nephropathy. The transplant itself was uneventful. His other past medical history consisted of transfusion dependent beta-thalassaemia intermedia (baseline ferritin was 2236 μg/l) and splenectomy. He was discharged on tacrolimus, MMF and prednisolone.

He was re-admitted 6 days later with a 2-day history of dry cough, dyspnoea and self-recorded pyrexia of 38°C. Examination revealed coarse crepitations predominantly on the right, saturation of 97% in room air, respiratory rate 18 breaths/min and blood pressure 161/84 mmHg. Blood tests showed a ferritin of 4054 μg/l, CRP 84 mg/l and procalcitonin of 1.71 μg/l. CXR showed consolidation in the right mid and lower zones, and NPS confirmed SARS-CoV-2. Ceftriaxone was initiated.

On day 2, his oxygen requirement increased, with worsening bilateral mid zone air space shadowing. He was commenced on continuous positive airway pressure 5 days after admission and underwent endotracheal intubation on day 7. Given the clinical deterioration and rising CRP (170 mg/l), he was commenced on anakinra 200 mg i.v. once a day (Table 1). After initiation of anakinra, inotropes were weaned within 24 h, and significant improvement in his blood parameters was noted (ferritin 2687 µg/l and CRP 26 mg/l).

His ICU admission was complicated by an *Enterococcus faecium* bacteraemia on day 9 of admission, for which he completed 7 days of teicoplanin. Anakinra was reduced and stopped after 21 days. After a successful tracheostomy wean, he was discharged on day 45.

### Case 3

A 68-year-old woman with a background of non-Hodgkin’s lymphoma was re-admitted 2 weeks after being discharged with COVID-19. She had known follicular lymphoma stage 4A diagnosed in May 2018. She was treated with rituximab, last receiving a dose 3 months before her first admission. At the time of her first admission, she presented with a few weeks’ history of fever, myalgia and a sore throat. She had been given co-amoxiclav and azithromycin in the community, 1 week before admission. Her shortness of breath on exertion was worsening, and she was found to have a saturation of 89%. NPS confirmed SARS-CoV-2, and her CXR was consistent with the diagnosis. She was managed on the ward with oxygen and i.v. antibiotics and was discharged 3 weeks later, having had a CT pulmonary angiogram, confirming no pulmonary emboli, and a PET scan, which showed no advancement of her non-Hodgkin’s lymphoma.

She was re-admitted 21 days later with profound hypoxia, requiring intubation on arrival in hospital. A CT pulmonary angiogram at the time showed extensive bilateral pulmonary emboli, with evidence of right heart strain. There were severe COVID-related changes, with widespread ground glass opacification throughout both lung fields. On admission, ferritin was 29 784 µg/l, CRP 78 mg/l and procalcitonin 2.04 μg/l. She was anaemic (haemoglobin 100 g/l), but not cytopenic, with platelets of 199 × 10^9^/l and a neurophilia of 15.9 × 10^9^/l. NPS and EDTA blood samples were positive for SARS-CoV-2.

She was admitted to the ICU, initiated on tazocin i.v. and underwent thrombolysis for her pulmonary emboli. The following day, ferritin rose to 40 069 µg/l, CRP was 109 mg/l, and procalcitonin increased to 9.85 μg/l. Anakinra i.v. was initiated (Table 1). She was initially started on 100 mg four times a day, but owing to pressures on the nursing staff this was changed to 200 mg twice a day. Within 24 h of commencing anakinra i.v., the ferritin improved to 20 479 μg/l (Fig. 1), and 3 days later continued to fall to 5118 μg/l. A BioFire film was positive for *Streptococcus* pneumonia. Her ICU stay was complicated by worsening consolidation on her CXRs, necessitating a prolonged course of meropenem. She commenced ambisome on day μg/l2 of admission, which was continued after a positive galactomannan test on her sputum and strongly positive βD-glucan (341.5 pg/ml), 2 weeks into her admission. It took 24 days from admission for the SARS-CoV-2 viraemia to disappear on both EDTA blood and NPS. She remains in the ICU and is currently weaning off the ventilator, with a tracheostomy *in situ* and on minimal inotropic support.

### Case 4

A 49-year-old woman presented with a 2-week history of non-productive cough and fever, with 1 week of diarrhoea. She had end-stage renal failure secondary to lupus nephritis, requiring haemodialysis. Her past medical history included APS with thromboses and ischaemic heart disease. Her medication included warfarin and prednisolone.

Initial blood tests showed an elevated CRP (339 mg/l) and ferritin (2890 µg/l), with bilateral patchy consolidation on CXR. She was admitted on oxygen 2 l/min via nasal cannula. Ceftriaxone and gentamicin were initiated, and SARS-CoV-2 was confirmed via NPS.

On day 6 her oxygen requirements increased, with worsening patchy consolidation bilaterally on CXR. She underwent endotracheal intubation in the ICU, and her antibiotics were changed to temocillin, teicoplanin and gentamicin.

On day 10, blood tests showed worsening thrombocytopenia and transaminitis with rising ferritin: platelets 91 × 10^9^/l, ferritin 7636 μg/l, with a peak in her procalcitonin of 198 μg/l. An assumed diagnosis of HLH was made, and she commenced anakinra 200 mg i.v. (Table 1). She required increasing inotropic support, and her thrombocytopaenia deteriorated, with associated increasing ferritin over the following days.

On day 17, blood tests revealed ferritin 30 086 μg/l. A CT chest revealed small bilateral lower lobe pulmonary emboli and extensive consolidation throughout both lungs. The clinical deterioration suggested worsening HLH; therefore, anakinra was increased sequentially to 300 mg twice a day, and antibiotics were switched to meropenem and caspofungin.

Two days later there was notable improvement in ferritin and CRP, and the transaminases started to normalize. The patient remains intubated and ventilated, with reducing inotropic support.

## Discussion

We present four cases of immunosuppressed patients, who received beneficial effects from the use of anakinra i.v. to treat severe COVID-19 with hyperinflammation and concomitant bacterial infections.

Careful consideration of the immunosuppressive treatment needs to factor in the ability to clear the virus without allowing for hyperinflammation. Anakinra has previously been used in the context of virally induced inflammatory conditions, such as multicentric Castleman’s disease, which is a reactive lymphoproliferative disorder typically described in HIV-positive patients, with a close association with HHV-8 [12].

Cavalli *et al.* [16] described the use of anakinra in patients with ARDS attributable to COVID-19 who also had hyperinflammation. They used anakinra at 5 mg/kg i.v. twice a day (high-dose group) for 21 days. A subgroup of patients received 100 mg s.c. twice a day (low-dose group) for 7 days. Standard care included 400 mg HCQ, along with lopinavir and ritonavir. At 21 days, survival was 90% in the high-dose group and 56% in the standard treatment group (*P* = 0.009).

Their lower threshold for diagnosis of hyperinflammation (CRP >100 mg/l or ferritin 900 ng/ml) and differing standard practice reflect a cohort of patients not necessarily classified as severe in the UK. A ferritin <500 ng/ml has a high negative predictive value for HLH [6], whereas >10 000 mg/l is diagnostic of HLH in children [17]. The use of oral HCQ might skew the mortality data, with a potential for increased cardiac complications [18]. An observational study on the use of HCQ in COVID-19 did not report any difference in mortality [19], and it is not included in UK treatment algorithms. Furthermore, concomitant bacterial infections were excluded.

Anakinra has received increasing use in the context of cytokine storm syndromes/HLH [4]. Although licensed s.c., concerns over unreliable absorption in the critically ill patient and the requirement of multiple injections, along with the support of its safety at higher doses in the context of sepsis, have all favoured i.v. administration. Support for our use of higher dose anakinra i.v. is also gained from the recent lack of benefit for the s.c. form in the context of COVID-19 [16].

The administration of anakinra i.v. leads to a maximal plasma concentration 24–29 times higher when compared with s.c. administration. It also has a shorter terminal half-life in the i.v. format (2.64 h, compared with 5.24 h s.c.) [20, 21], with the caveat that the s.c. half-life increases with greater adipose tissue. The i.v. form therefore enables a higher and faster maximal plasma concentration of anakinra; a trait that is desirable during cytokine storm syndromes.

There is currently not sufficient evidence to suggest that the majority of patients with severe COVID-19 develop HLH, because the other diagnostic parameters not including ferritin (hypertriglyceridaemia, low fibrinogen and cytopenias) are not correlated with severe disease [22]. None of our cases developed organomegaly, and only case 4 showed evidence of thrombocoytopenia, but the other blood count lineages did not fall. Therefore, we believe we are treating hyperinflammation and not HLH in our four cases.

The timing of treatment with anakinra is one of the challenges in managing patients with COVID-19 and of any clinical trial design. Although antiviral therapy is likely to be beneficial in the early phase of the disease, cytokine modulation and immunosuppressive therapy are most likely to have an impact at later stages [11]. A number of trials are attempting to answer this question. A recent large adaptive-platform trial (Recovery trial) showed a significant benefit on mortality of low-dose dexamethasone, with the biggest reduction in risk of mortality being observed in patients requiring invasive mechanical ventilation [23]. This affirms the concept of the need for immune modulation in critically unwell patients requiring ventilatory and organ support. Further trials in the UK include REMAP-CAP (https://www.remapcap.org/), an adaptive-platform trial to assess the use of antibiotics, antivirals and immunomodulators in COVID-19. It offers the opportunity to explore targeted immune modulation therapy, which will include IFN-β1a, IL-1 receptor antagonists (anakinra), tocilizumab and sarilumab (both anti-IL-6), all against placebo.

COVACTA (https://clinicaltrials.gov/ct2/show/NCT04320615) randomized severe SARS-CoV-2 pneumonitis patients to tocilizumab (a monoclonal antibody targeting the IL-6 receptor). CAN-COVID (https://clinicaltrials.gov/ct2/show/NCT04362813) randomizes canikunimab (a monoclonal antibody that targets the IL-1β cytokine specifically) to patients with severe COVID-19, not requiring mechanical ventilation.

The results of these trials will be extremely informative to direct treatment strategy for COVID-19 infections in the future. Whether single treatment, combination therapy or early intervention is optimal remains to be elucidated.

Our case series supports the hypothesis of IL-1 blockade as an important disease-modifying treatment in those patients with severe, late-stage COVID-19, with evidence of cytokine storm. We show that the i.v. form is safe at high doses, even in patients with concomitant bacterial infections. We believe that administering anakinra i.v. at the height of the cytokine storm has profound beneficial effects, both clinically and biochemically, on patients with severe COVID-19 infection.

*Funding:* No specific funding was received from any bodies in the public, commercial or not-for-profit sectors to carry out the work described in this manuscript.

*Disclosure statement:* The authors have declared no conflicts of interest.
